# A Microbial Perspective on the Grand Challenges in Comparative Animal Physiology

**DOI:** 10.1128/mSystems.00146-17

**Published:** 2018-03-06

**Authors:** Kevin D. Kohl

**Affiliations:** a Department of Biological Sciences, University of Pittsburgh, Pittsburgh, Pennsylvania, USA

**Keywords:** animal physiology, host-microbe interactions, organismal biology

## Abstract

Interactions with microbial communities can have profound influences on animal physiology, thereby impacting animal performance and fitness. Therefore, it is important to understand the diversity and nature of host-microbe interactions in various animal groups (invertebrates, fish, amphibians, reptiles, birds, and mammals). In this perspective, I discuss how the field of host-microbe interactions can be used to address topics that have been identified as grand challenges in comparative animal physiology: (i) horizontal integration of physiological processes across organisms, (ii) vertical integration of physiological processes across organizational levels within organisms, and (iii) temporal integration of physiological processes during evolutionary change. Addressing these challenges will require the use of a variety of animal models and the development of systems approaches that can integrate large, multiomic data sets from both microbial communities and animal hosts. Integrating host-microbe interactions into the established field of comparative physiology represents an exciting frontier for both fields.

## Abstract (precis)

Interactions with microbial communities can have profound influences on animal physiology, thereby impacting animal performance and fitness. Therefore, it is important to understand the diversity and nature of host-microbe interactions in various animal groups (invertebrates, fish, amphibians, reptiles, birds, and mammals).

## PERSPECTIVE

Aspects of animal physiology and performance are important considerations in ecology and evolution, as improved animal performance will promote an animal’s fitness (1). Recently, a great deal of research has demonstrated the important role that host-microbe interactions play in the physiology and performance of animals. These findings have given rise to the notion of the “holobiont,” stating that natural selection acts on the collective of animal hosts plus all of their stable and transient microbes (2). Given these ideas, it is becoming increasingly important to understand the connections between host-associated microbes, physiological performance, and animal fitness in natural populations.

In 2010, the Society for Integrative and Comparative Biology (SICB) hosted a workshop focusing on identifying the grand challenges in organismal biology and subsequently solicited publications identifying the grand challenges in subdisciplines of organismal biology. The field of comparative physiology utilizes the functional diversity of organisms to study how physiological systems allow animals to perform particular functions and may be adapted to particular environments and is closely linked to the fields of environmental physiology and evolutionary physiology. The grand challenges in comparative physiology were identified by Mykles et al. (3) as (i) horizontal integration of physiological processes across organisms within ecosystems, (ii) vertical integration of physiological processes across organizational levels within organisms, and (iii) temporal integration of physiological processes during evolutionary change. Given that host-associated microbes can strongly influence animal physiology, it is important to consider how these interactions should be incorporated into the field of comparative physiology and especially for addressing these grand challenges.

In this perspective, I will discuss how the field of host-microbe interactions can be incorporated into comparative physiology in a way that addresses the grand challenges of the field. In the 2010 paper (3), Mykles et al. explicitly discuss host-microbe interactions as a rich area for studying horizontal integration of physiological processes across organisms within ecosystems. The challenge of vertical integration across levels of biological organization exists in the field of comparative physiology but is also becoming increasingly important for understanding host-microbe interactions as multiomics approaches become more common in this field. Temporal integration of host-microbe interactions and animal physiology will be an exciting area to study phenotypic plasticity and the role of individual variation, two large challenges in the field of comparative physiology. Last, I will briefly highlight some concluding thoughts and paths forward to advance these fields.

## HORIZONTAL INTEGRATION ACROSS ORGANISMS AND VERTICAL INTEGRATION ACROSS LEVELS OF BIOLOGICAL ORGANIZATION

**FIG 1:**
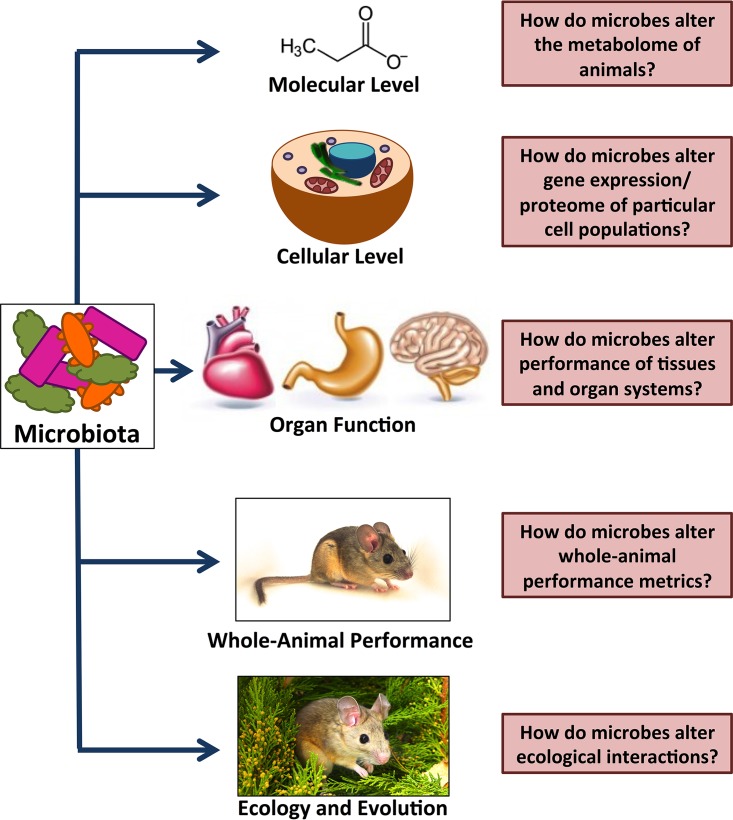
Numerous levels of biological organization can be affected by animal–microbe symbioses. Addressing questions at each of these levels and the integration across them will greatly enhance our understanding of animal–microbe symbioses. (Modified from reference 19 with permission.)

The field of comparative physiology can be studied at many levels of biological organization, such as molecules and genomes, to tissues and organs, up to complex traits such as behavior and whole-animal metabolism. The inclusion of data from microbial communities (e.g., microbial inventories, metagenomics, and activities of microbial enzymes) will increase our understanding of these systems and their interactions (Fig. 1). In comparative physiology, a definitive way to test for genome-phenotype or genome-physiology integration is through reverse genetics (3). An equivalent experimental technique in the field of host-microbe interactions might be microbiome removal (the use of germfree animals) or microbial transplantation across animal hosts. These techniques have been widely used to understand the role of the microbiome in human physiology (4, 5) and will become increasingly important for understanding microbial contributions to host physiology.

My research program has utilized microbial transplants to test for the horizontal integration between organisms. For example, I have studied how gut microbial communities aid herbivorous mammals in the detoxification of plant defensive compounds. This work has focused on woodrats (*Neotoma* spp.), small herbivorous rodents that tend to be dietary specialists of toxic plants (6). Woodrats are especially interesting, as different species and populations specialize on plants containing distinct defensive compounds and seem to be particularly adapted to the toxins in their natural diet, allowing for comparative and experimental studies (6). We have conducted microbial transplants from toxin-adapted populations of woodrats into naive recipients (populations where the toxic plant does not cooccur with the woodrats) to demonstrate that the gut microbiome is important in allowing herbivores to consume plant toxins (7). We also took an even more reductive approach where we cultured individual microbes capable of degrading tannins (a chemical class of plant toxins) and inoculated these microbes into recipient lab rats (8). Lab rats that received an inoculation of tannin-degrading bacteria were able to consume higher doses of tannins, demonstrating that the interactions between these organisms are important for feeding on toxins. I have also conducted microbial transplants from several species of* Peromyscus* mice into a single focal species (*Peromyscus polionotus*). Recipient mice that received microbial transplants from more distantly related hosts exhibited a decreased ability to digest food material (9). Further, *Nasonia* wasps inoculated with the microbiota from other species exhibited decreased growth and survival compared to larva inoculated with intraspecific transplants (9). These results demonstrate horizontal integration between hosts and microbes and show that hosts have an optimal or compatible gut microbial community, with which animal performance is improved.

My research program has also addressed host-microbe interactions at various levels of biological organization. When feeding on diets containing plant defensive compounds, the woodrat gut microbiome shifts in community structure (10) and functions (7), results that were demonstrated by 16S rRNA inventories and metagenomics, respectively. Further, we have investigated the numerous physiological effects of microbial transplants in allowing herbivores to consume plant toxins (7). At low doses of toxins, animals that received microbial transplants from toxin-adapted woodrats were able to maintain body mass, while control animals lost body mass (7). Interestingly, treatment groups did not differ in their food intakes or abilities to digest food, and so these mechanisms did not underlie the differences in body mass (7). Therefore, we used a metabolomics approach and found that microbial transplantation altered the signatures of toxin metabolites in the urine of woodrats (7) and reduced indicators of liver damage (8), demonstrating that gut microbes altered detoxification pathways. Therefore, microbial transplants likely alleviate the metabolic costs of hepatic detoxification. Finally, these physiological effects scale up to influence whole-animal performance: hosts receiving microbial transplants consume higher doses of plant toxins and maintain body mass when placed on toxic diets (7, 8). It is only through this multifaceted research approach that we could uncover some of the physiological mechanisms promoting consumption of plant toxins.

## TEMPORAL INTEGRATION OF PHYSIOLOGICAL PROCESSES DURING EVOLUTIONARY CHANGE

Both host-microbe interactions and aspects of physiological performance have been identified as important factors in the natural selection of animals (1, 2). One prediction of the hologenomic theory of evolution is “phylosymbiosis,” which hypothesizes that microbial communities should be more similar within a host species than across species and that increasing genetic divergence between host species will be associated with greater differences in their microbial communities (2). Indeed, we have demonstrated concordance between the evolutionary history of host species and dendrograms of the similarities in host-associated microbial community structures (9). Additionally, given that interspecific microbial transplants yielded decreases in performance and survival (as described above), these trends have functional consequences that influence host fitness.

One challenge associated with temporal integration of physiology processes is phenotypic flexibility, or how the physiological capabilities can vary depending on the environment. The gut microbiome is also known to respond rapidly to environmental changes such as diet, ambient temperature, and salinity. Presumably, these changes also yield changes in microbiome function and therefore affect host physiology. Cold-acclimated mice exhibit longer intestines to maximize digestion and absorption of nutrients, and microbial transplants confer these physiological effects on germfree mice held at room temperature (11). The gut microbiota of lizards responds to changes in diet, and some aspects of microbial community structure, such as the relative abundances of sulfate-reducing bacteria, correlate with the ability of lizards to digest fiber material (12). The physiological flexibility conferred by gut bacteria is likely important for animal fitness, though establishing this link still requires more research.

Another important aspect of evolutionary physiology is the existence of individual variation. Many physiological values, such as metabolic rate, hormone concentrations, or locomotor performance, exhibit individual variation, and there has been a great deal of research aimed at understanding how individual variation links to animal fitness. Host-associated microbial communities are also known to exhibit strong individual variation across hosts. In a long-term, repeated, artificial selection experiment, voles were selected for the ability to maintain body mass when placed on a high-fiber diet (13). After 13 generations, voles in the selected lines exhibited microbial community structures distinct from those of randomly bred, control lines. This result suggests a connection between individual variation in microbial community composition and individual variation in the ability to cope with high-fiber diets. There has been a call for additional experimental evolution systems to better understand the evolution of beneficial host-microbe interactions (14). It would also be interesting to study the role of individual variation in our woodrat system. We have plans to search for correlations between gut microbial community structure and the ability of woodrats to tolerate high-toxin diets (measured as voluntary intake or ability to maintain body mass).

## FUTURE DIRECTIONS

The field of comparative physiology largely subscribes to an idea known as Krogh’s principle, which states that “for such a large number of problems there will be some animal of choice, or a few such animals, on which it can be most conveniently studied” (15). This idea can also apply to host-microbe interactions, such that toxin-adapted woodrats are an ideal study system for investigating microbial detoxification (6). Similarly, we have gained a great deal of knowledge of host-microbe interactions from invertebrate models such as the Hawaiian bobtail squid (16), *Hydra* (17), and other animals. The time is now ripe for biologists to study host-microbe interactions and their physiological consequences in a wide variety of animal models. This is even more possible now as genomes of microbes and animal hosts are becoming more widely available and the costs of conducting multiomics approaches in nonmodel systems are decreasing.

Integration of multiomics data across many levels of biological organization of both microbes and hosts will undoubtedly require complex analyses. Quantitative systems biology uses models to establish links between these levels of biological organization and will be an important feature of research in this area. However, it should also be recognized that generating several types of omics data can be prohibitively expensive, especially for most comparative physiology research groups. There is still great utility in more-traditional physiological techniques, such as enzyme assays and basic physiological measurements. Similarly, on the microbiota side, there has been a call for a return to culturing techniques to understand the microbial diversity. Microbial communities can be easily manipulated, through antibiotics, microbial transplants, or selection using nutrient-deprived/rich growth media (18). The combination of multiomics systems approaches and more-traditional research techniques will push forward the fields of comparative physiology and host-microbe interactions.
